# Up-Conversion Luminescence and Optical Temperature Sensing Properties of NaLuF_4_:Yb^3+^/Ho^3+^ Micron-Sized Crystals at Low Temperature

**DOI:** 10.3390/nano14151292

**Published:** 2024-07-31

**Authors:** Tian Zhang, Zhaojin Wang, Jin Hou, Xinyi Xu, Xin Zhao, Zijie Li, Siyi Di

**Affiliations:** 1 Institute of Physics and Optoelectronics Technology, Baoji University of Arts and Sciences, Baoji 721016, China; zhangtian@stu.bjwlxy.edu.cn (T.Z.); houjin@stu.bjwlxy.edu.cn (J.H.); xuxinyi@stu.bjwlxy.edu.cn (X.X.); zhaoxin@stu.bjwlxy.edu.cn (X.Z.); lizijie@stu.bjwlxy.edu.cn (Z.L.); disiyi@stu.bjwlxy.edu.cn (S.D.); 2 Baoji Ultrafast Lasers and Advanced Materials Science and Technology Center, Baoji 721016, China

**Keywords:** up-conversion luminescence, thermally coupled and non-thermally coupled energy levels, fluorescence intensity ratio, dual mode, low temperature, optical temperature sensing

## Abstract

Non-contact temperature sensors utilising the fluorescence intensity ratio and the unique up-conversion (UC) luminescence of rare-earth ions have numerous benefits; however, their operational temperature range has remained limited. In this study, NaLuF_4_:Yb^3+^/Ho^3+^ samples were prepared by the hydrothermal method. The samples exhibited exceptional UC luminescence properties at low temperatures. The intensity of the green emission (with peak wavelengths of 540 and 546 nm) gradually decreased with increasing temperature, and the green emissions showed a unique change at low temperatures. In addition, we studied the dependence of the UC luminescence intensity on the excitation power and the variation in the decay lifetime with temperature. The experiments revealed excellent luminous performance and significantly enhanced sensitivity at low temperatures; the maximum absolute sensitivity S*_a_* and relative sensitivity S*_r_* of the 540 and 546 nm thermally coupled energy levels were 1.02% and 0.55% K^−1^, respectively. The potential temperature sensing properties of Yb^3+^/Ho^3+^-co-doped NaLuF_4_ makes it suitable for temperature sensing applications at temperatures as low as 30 K. This study offers a novel approach for the advancement of temperature sensing technology at low temperatures.

## 1. Introduction

Through their unique up-conversion (UC) behaviour, lanthanide compound micron-sized crystals convert low-energy radiation to high-energy radiation. They possess numerous distinct benefits in the fields of electronics, optical devices, colour displays, and biomedical imaging [1,2,3,4]. Recently, the development of fluorescent temperature sensors utilising the fluorescence intensity ratio (FIR) and the UC luminescence of rare-earth ions has garnered significant interest and research efforts. In the field of temperature measurement, non-contact temperature sensing technology utilising rare-earth ion–doped luminescent materials has garnered significant interest, primarily because of its potential utility in the field of electric power [5,6,7]. Compared with conventional thermocouple or thermal resistance measurement methods, optical temperature measurement is non-contact, highly accurate, and rapidly responsive. This offers new possibilities in the field of temperature monitoring [8].

Liu et al. studied temperature sensing based on Yb^3+^/Ho^3+^-co-doped Gd_2_O_3_ and FIR technology at 130–280 K and obtained a maximum relative sensitivity S*_r_* of 0.80% K^−1^ at 216 K [9]. In 2021, Li et al. researched the temperature sensing performance of LiYF_4_:Yb^3+^/Ho^3+^ in the temperature range of 100–500 K and obtained a maximum absolute sensitivity S*_a_* of 4.77% K^−1^ and maximum S*_r_* of 1.29% K^−1^ [10]. In the same year, Singh et al. studied temperature sensing based on the thermally coupled energy levels of Ho^3+^ in the temperature range of 305–515 K, and found a maximum S*_r_* of 0.34% K^−1^ at 305 K [6]. Recently, Zhang et al. evaluated Gd*_x_*Y_1−*x*_TaO_4_ (GYTO):Yb^3+^/Ho^3+^ temperature sensing at 330–660 K and obtained a maximum S*_a_* of 0.1% K^−1^ and maximum S*_r_* of 0.37% K^−1^ [11]. In 2022, Gao et al. studied the red emission enhancement of Ce^3+^-doped NaLuF_4_:Yb^3+^/Ce^3+^/Ho^3+^@NaLuF_4_:Yb^3+^/Nd^3+^. They mainly investigated the UC mechanism and found that the red emission enhancement can be regulated by introducing Ce^3+^ [12]. Zhou et al. studied the temperature sensing characteristics of Ce^3+^-doped NaLuF_4_:Yb^3+^/Ho^3+^ in the temperature range of 30–300 °C. They found that as the concentration of Ce^3+^ ions increased, the absolute sensitivity escalated from 0.36% to 4.37% K^−1^ [13]. However, the temperature measurement range remained limited. Thus, this study extended the temperature range down to 30 K using Yb^3+^/Ho^3+^-co-doped NaLuF_4_. Based on FIR and luminescence intensity ratio (LIR) technology, the thermal and non-thermal coupling effects on the temperature sensing properties of Ho^3+^ were investigated.

## 2. Materials and Methods

### 2.1. Experimental Materials

In this experiment, the primary materials included trisodium citrate (purity 99%), NH_4_F (purity 96%), and RE(NO_3_)_3_•6H_2_O (RE = Lu, Yb, Ho) (purity 99.99%). The chemical reagents used in this study were purchased from China National Pharmaceutical Chemical Reagent Co., Ltd. (Shanghai, China). All experiments were conducted using deionised water, and all chemical samples were used as-is.

### 2.2. Sample Preparation

Sodium citrate was used as a stabiliser and chelating agent, and the hydrothermal method was used to synthesise NaLuF_4_:20%Yb^3+^/2%Ho^3+^ micron-sized crystals. Trisodium citrate (0.5884 g) was mixed with 30 mL of deionised water in a 100 mL beaker and stirred for 20 min on a magnetic mixer. A certain proportion of RE(NO_3_)_3_•6H_2_O aqueous solution with concentration of 0.5 mmol/cm^3^ (0.2 mL Yb(NO_3_)_3_, 20 μL Ho(NO_3_)_3_, 780 μL Lu(NO_3_)_3_ solution) was added and stirred well, and then 0.2960 g of NH_4_F was added. The stirring continued until complete dissolution. The solution obtained was introduced into a 50 mL reactor, and the temperature of the reaction stove was set at 200 °C for 24 h. At the end of the reaction, the system was allowed to cool down to ambient temperature before extracting the sample. The sample was rinsed multiple times with ethanol and deionised water, centrifuged to collect the precipitate, and dried at 80 °C for 6 h. Finally, NaLuF_4_:20%Yb^3+^/2%Ho^3+^ was collected.

### 2.3. Analytical Methods

The crystal structures of the samples were analysed using powder X-ray diffraction (XRD) at room temperature. The wavelength was 0.15405 nm, the target was copper K-α, the tube voltage was 30 kV, and the tube current was 10 mA. The angle range was 5°–80°, and the step size was 0.02°. The microstructures of the samples were examined using scanning electron microscopy (SEM; TESCAN VEGA 3 scanning electron microscope, Brno, Czech Republic). Element mapping (EM) of the samples was performed using SEM. The emission spectra and luminescence lifetimes of the samples were measured using a fluorescence spectrometer (FLS-980, Edinburgh Company, Edinburgh, UK) with Low Temperature Annex (Optistant Dry, Oxford, UK) and High Temperature Annex (TCB1402C, Shanghai Tianmei Technology Co., Shanghai, China). The excitation light source was a 980 nm semiconductor laser with pulse modulator (Edinburgh Company, UK).

## 3. Results and Discussion

### 3.1. Sample Characterisation

**Figure 1:**
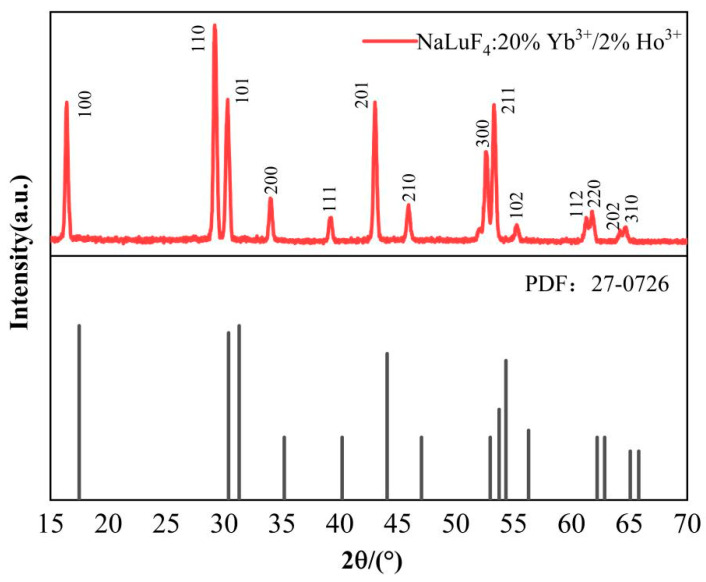
X-ray diffraction (XRD) pattern of prepared NaLuF_4_:20%Yb^3+^/2%Ho^3+^ micron-sized crystal and β-NaLuF_4_ standard pattern.

Figure 1 shows the XRD pattern of the NaLuF_4_:20%Yb^3+^/2%Ho^3+^ micron-sized crystals. As shown in the characterisation diagram, the diffraction positions of the sample coincided with each peak of the standard card (PDF #27-0726). The diffraction peaks were sharp, no other diffraction peaks were observed, and the crystal quality of the sample was satisfactory. Notably, we prepared Yb^3+^/Ho^3+^-co-doped NaLuF_4_ micron-sized crystal particles with hexagonal phases. The data presented in Figure 1 show that the diffraction peaks of the samples shifted noticeably towards smaller angles. This is because the ionic radii of Ho^3+^ (0.089 nm) and Yb^3+^ (0.086 nm) were larger than that of Lu^3+^ (0.085 nm). When the Ho^3+^ and Yb^3+^ ions replaced the Lu^3+^ ions, lattice expansion resulted in a shift in the diffraction peaks towards smaller angles.

**Figure 2:**
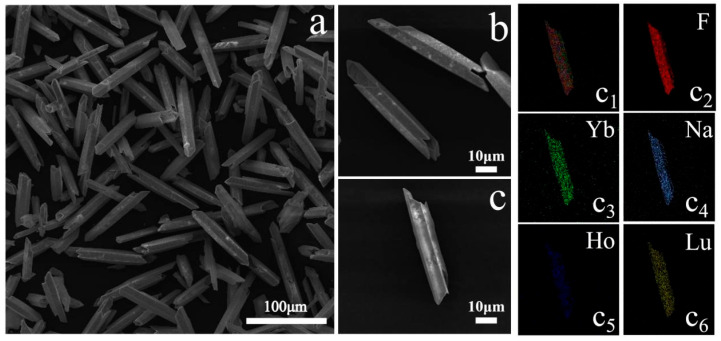
(**a**,**b**) Scanning electron microscopy (SEM) images; (**c**) Element mapping (EM) of prepared NaLuF_4_:20%Yb^3+^/2%Ho^3+^ micron-sized crystal sample. (**c_1_**) The doping elements distribution of NaLuF_4_:20%Yb^3+^/2%Ho^3+^; (**c_2_**–**c_6_**) corresponding to elements F, Yb, Na, Ho, and Lu, respectively.

Figure 2a,b show SEM images of the NaLuF_4_:20%Yb^3+^/2%Ho^3+^ micron-sized crystals. The crystals are evidently uniform; they are hollow cylinders that are smooth in the middle and sharp at both ends, and they have a length of approximately 80 μm, width of approximately 10 μm, and thickness of approximately 60 nm. Figure 2c presents the EM of the sample, which shows the distribution of each doped element; only the target elements were present. The results show that NaLuF_4_:Yb^3+^/Ho^3+^ micron-sized crystals were successfully prepared.

### 3.2. UC Luminescence Properties at High and Low Temperatures

**Figure 3:**
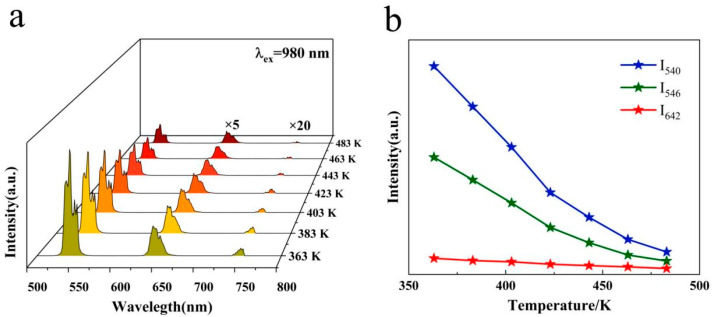
(**a**) Fluorescence spectra of samples at high temperatures under 980 nm laser excitation; (**b**) variation in intensity with temperature for samples at 540, 546, and 642 nm.

The NaLuF_4_:20%Yb^3+^/2%Ho^3+^ was excited by a 980 nm near-infrared laser. Figure 3a shows the UC luminescence spectra at high temperatures, revealing three emission peaks at 540, 642, and 750 nm, where the red and near-infrared bands were magnified by factors of 5 and 20, respectively, to enhance the visibility of its trend. The wavelengths at which the green and red emission peaks occurred remained essentially constant as the temperature increased, and the green emission intensity decreased sharply, whereas that of the red decreased slowly. Figure 3b shows the UC intensity and temperature changes at 540, 546, and 642 nm. Clearly, the intensity of green and red emissions decreased with increasing temperature. G1 (540 nm) is corresponding to ^5^F_4_→^5^I_8_, and G2 (546 nm) is corresponding to ^5^S_2_→^5^I_8_. Notably, under a 980 nm laser stimulation, the green emission was intense, and the intensity of G1 was markedly greater than that of G2.

**Figure 4:**
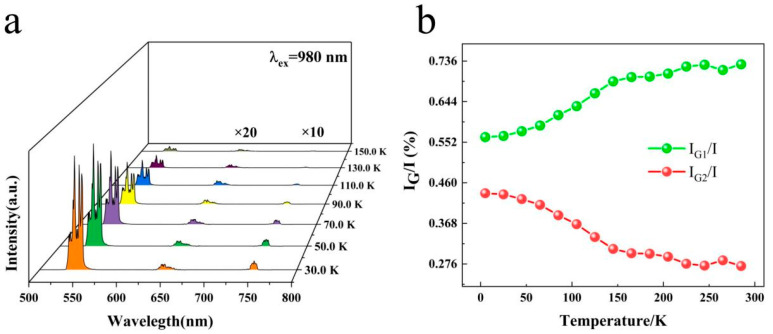
(**a**) Luminescence spectra at low temperatures; (**b**) integral area ratio for 540 nm (^5^F_4_) and 546 nm (^5^S_2_).

Figure 4a shows the fluorescence spectra at low temperatures, where the red and near-infrared bands were magnified by factors of 20 and 10, respectively, to more clearly observe the trend of changes. The spectra reveal three emission peaks at 540, 642, and 750 nm. As the temperature was increased from 30 to 150 K, the variation trend of intensity remained consistent with that of high temperatures. The emission peaks did not shift with increasing temperature; however, their magnitude decreased, the green emission intensity decreased significantly, whereas the red emission intensity diminished at a slower pace. The red emissions were extremely weak and thus hard to lay out at low temperatures. Figure 4b shows G1 and G2, mirroring the ratio of the integral area to the entire green luminescence integral area. Their integration ranges were 520–545 and 545–570 nm, respectively. With increasing temperature, the proportion of G1 increased, and that of G2 decreased.

### 3.3. UC Luminescence Mechanism

**Figure 5:**
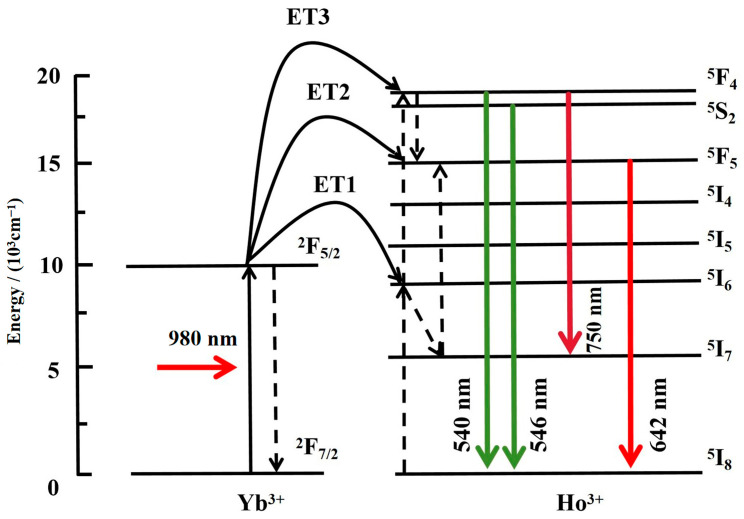
Energy level diagram of Yb^3+^ and Ho^3+^ ions and up-conversion (UC) mechanism.

The energy exchange process between the rare-earth ions, Yb^3+^ and Ho^3+^, was explored (Figure 5). The 980 nm radiation stimulated Ho^3+^ and Yb^3+^ through ground state absorption (GSA). Because the absorption cross-section of Ho^3+^ ions was restricted, the GSA process was significantly weak against Ho^3+^, and the absorption cross-section of Yb^3+^ at a wavelength of 980 nm was approximately an order of magnitude greater than that of Ho^3+^. In this material, the concentration of Yb^3+^ was 10 times that of Ho^3+^, and most incident photons were absorbed by the Yb^3+^ ions, which transferred energy to the Ho^3+^ ions through an energy-transfer mechanism.

The UC luminescence mechanism is as follows: the Yb^3+^ ion at the ^2^F_7/2_ level absorbs the energy emitted by the 980 nm laser and transitions to the ^2^F_5/2_ level. Yb^3+^ then sensitises Ho^3+^ through non-resonant energy transfer involving phonons, and Ho^3+^ at the ^5^I_8_ level transitions to ^5^I_6_. Some of the ions first reach the ^5^I_7_ level by a non-radiative transition and then undergo the nearby Yb^3+^ energy transition to the ^5^F_5_ level, returning to the ground state and emitting red luminescence. Other ions can accept the nearby Yb^3+^ energy transition to the ^5^F_4_/^5^S_2_ level, and some of these ions can radiate to the ^5^I_7_ level and ground state, emitting near-infrared and green luminescence, respectively. The remaining particles, which reside at the ^5^F_4_/^5^S_2_ level, undergo a non-radiative transition to the ^5^F_5_ level, returning to the ground state and emitting red luminescence. This is the main population responsible for red luminescence. The 540, 546, 642, and 750 nm UC luminescence emissions of NaLuF_4_:Yb^3+^/Ho^3+^ micron-sized materials result from the ^5^F_4_→^5^I_8_, ^5^S_2_→^5^I_8_, ^5^F_5_→^5^I_8_, and ^5^F_4_/^5^S_2_→^5^I_7_ transitions, respectively. Owing to the large energy gap of ^5^I_6_, ^5^I_7_ and ^5^F_4_/^5^S_2_, ^5^F_5_ levels, multiple-phonon assistance is required to complete the non-radiative relaxation (^5^F_4_/^5^S_2_→^5^F_5_ and ^5^I_6_→^5^I_7_), and the relaxation probability is small. The ^5^I_6_→^5^I_7_ and ^5^F_4_/^5^S_2_→^5^F_5_ non-radiative relaxations are very weak; consequently, the ^5^F_5_ and ^5^I_7_ levels of Ho^3+^ are difficult to populate, which leads to weak red emission. At low temperatures, both the lattice vibration and non-radiative relaxation become weaker, resulting in an almost imperceptible red emission.

**Figure 6:**
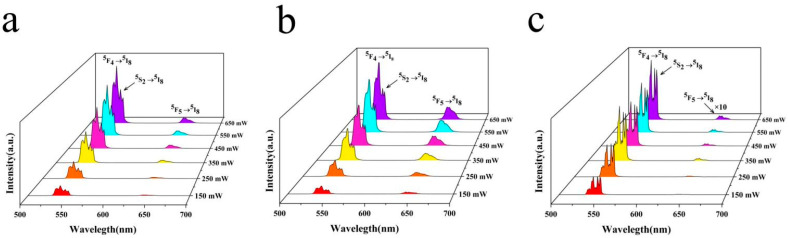
Sample UC luminescence spectra for different excitation powers at (**a**) 303 K, (**b**) 483 K, and (**c**) 4 K.

To better clarify the process behind the UC luminescence, we studied the relationship between the green and red luminescence intensities of Ho^3+^ under different excitation powers. Figure 6a–c shows the UC spectra of fluorescent Yb^3+^/Ho^3+^-co-doped NaLuF_4_ materials under different 980 nm laser power density excitations at temperatures of 303, 483, and 4 K. The red emission spectrum was magnified by a factor of ten at 4 K to facilitate the observation of trends. The data depicted in Figure 6 illustrate that under 980 nm laser excitation at 303, 483, or 4 K, the UC luminescence intensity of the sample gradually increased as the excitation power was increased from 150 to 650 mW, whereas the shape of the spectrum and the wavelength corresponding to the peak value did not change or shift significantly. The UC spectrum of the sample mainly included the following two emission bands: The green emission resulted from the ^5^F_4_/^5^S_2_→^5^I_8_ transition, with a wavelength range of 520–570 nm and a consuming emission signal corresponding to peak wavelengths of 540 and 546 nm. The red emission produced by the ^5^F_5_→^5^I_8_ transition corresponded to a wavelength range of 620–660 nm, and the emission signal was weak, corresponding to a peak wavelength of 642 nm.

**Figure 7:**
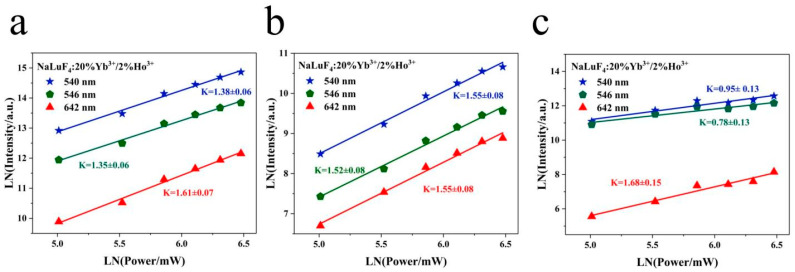
Double-log diagram at 540, 546, and 642 nm at (**a**) 303 K, (**b**) 483 K, and (**c**) 4 K.

Under unsaturated excitation, the UC luminescence intensity *I* generated by the transition from the excited state level to the lower level is related to the pump power *P* in accordance with the following [14]:

(1) $$I\propto P^{n},$$

taking the natural logarithm of both sides of Equation (1) yields the following:

(2) LnI∝nLnP,

where *n* denotes the number of photons requiring the absorption of 980 nm near-infrared radiation to stimulate the UC luminescence process. For UC luminescence, a logarithmic plot of the luminescence intensity and power density at different wavelengths represents the number of photons captured during a specific UC process. Figure 7a–c show the correlation between the logarithmic intensity and logarithmic pump power at 540, 546, and 642 nm at 303, 483, and 4 K, respectively. The slopes at 540, 546, and 642 nm are 1.38, 1.35, and 1.61 at 303 K; 1.55, 1.52, and 1.55 at 483 K; and 0.95, 0.78, 1.68 at 4 K, respectively. It is a two-photon UC process at both high and low temperatures. At ultra-low temperatures, the non-radiative relaxation is inhibited, and the luminous efficiency of the sample is high. The population conforms to Boltzmann’s law, the high-level population is more easily saturated at low temperature, and the saturation effect occurs [15,16]. Therefore, the slope is around one at ultra-low temperature. Analysis of the variable temperature spectrum indicates that the intensity of the red emission remained largely unaffected by temperature changes. In conclusion, the red emission demonstrates a low sensitivity to temperature variations, whereas the green emission exhibits a pronounced temperature response.

### 3.4. Relationship between Decay Lifetime and Temperature

**Figure 8:**
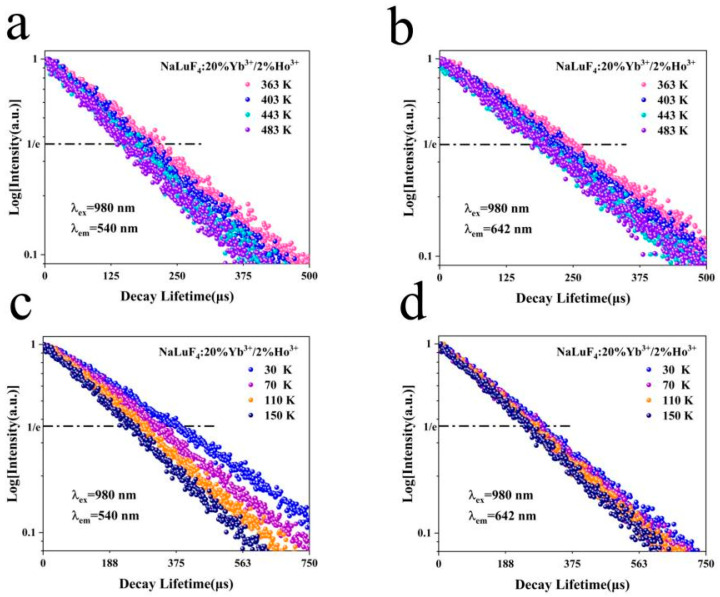
Decay lifetime at high temperatures at (**a**) 540 and (**b**) 642 nm; Decay lifetime at low temperatures at (**c**) 540 and (**d**) 642 nm.

To further investigate the UC luminescence characteristics, lifetime measurements of the UC luminescence by a 980 nm laser, and exponential fitting was performed. The fitting function is as follows [17]:

(3) $$y=y_{0}+A_{1}e^{-x/t_{1}},$$

where *A*_1_ is a constant and *t*_1_ is the decay lifetime. The emission peak lifetimes of the system are shown in Figure 8a,b. The correlation between the lifetime of the UC luminescence and temperature can be deduced through an exponential analysis of the emission peak data. As the temperature increased from 363 to 483 K, the decay lifetime dropped from 195.6 to 157.3 μs at 540 nm and from 230.1 to 186.9 μs at 642 nm. The emission peak lifetimes of the system at low temperatures are shown in Figure 8c,d. Upon a decrease in temperature from 150 to 30 K, the decay lifetime increased from 238.5 to 388.9 μs at 540 nm and from 256.3 to 291.1 μs at 642 nm.

These measurements indicate that the UC luminescence lifetime progressively shortens as the temperature rises. The lifetime decay formula is as follows [18]:

(4) $$\tau =\frac{1}{\Gamma_{\mathrm{rad}}+k_{\mathrm{nr}}},$$

where τ is the decay time, $\Gamma_{\mathrm{rad}}$ is the radiative transition probability, and $k_{\mathrm{nr}}$ is the non-radiative transition probability. As the temperature increased, the decay lifetime decreased. Conversely, as the temperature decreased, the decay lifetime increased. Regardless of whether the temperature increased from low temperature to room temperature or from room temperature to high temperature, the decay lifetime progressively shortened, and the non-radiative relaxation increased with increasing temperature. The temperature-induced changes in the decay lifetimes reflect the interplay between radiative and non-radiative transitions. The non-radiative transition probability increased with increasing temperature, leading to temperature quenching. The excited state energy dissipated as heat, resulting in a gradual decrease in UC luminescence intensity.

### 3.5. Optical Temperature Sensing at High and Low Temperatures

The emissions at 540/546 nm (^5^F_4_/^5^S_2_→^5^I_8_) and 540/642 nm (^5^F_4_/^5^F_5_→^5^I_8_) exhibited a clear temperature dependence. Notably, at low temperatures, the UC luminescence intensity of the red band, resulting from the ^5^F_5_→^5^I_8_ transition, changed a little. This stability could be attributed to the cooling effect, which suppresses multi-phonon relaxation, thereby reducing the population of ^5^F_5_ and leading to minimal temperature-induced variations. At room and high temperatures, the luminescence intensity differed significantly between G1 and G2. Significantly, at low temperatures, the proportions of G1 and G2 were roughly equal. However, as the temperature increased, the proportion of G1 steadily increased, whereas that of G2 correspondingly decreased. This shift indicates an increase in the high-energy-level population of G1, and the thermally coupled energy levels (^5^F_4_/^5^S_2_) demonstrated a notable temperature sensitivity.

The levels are commonly divided into thermally coupled and non-thermally coupled based on the FIR. The energy level difference is 200–2000 cm^−1^ for the thermally coupled levels and larger than 2000 cm^−1^ for the non-thermally coupled levels [19]. These levels are thermally coupled to the Boltzmann distribution law. The energy level separation between the ^5^F_4_ and ^5^S_2_ states is approximately 180 cm^−1^ [20]. The energy level interval between ^5^F_4_ and ^5^F_5_ is approximately 3000 cm^−1^, the levels ^5^F_4_ and ^5^S_2_ of Ho^3+^ are thermally coupled, and the ^5^F_4_ and ^5^F_5_ levels belong to a set of non-thermally coupled levels.

**Figure 9:**
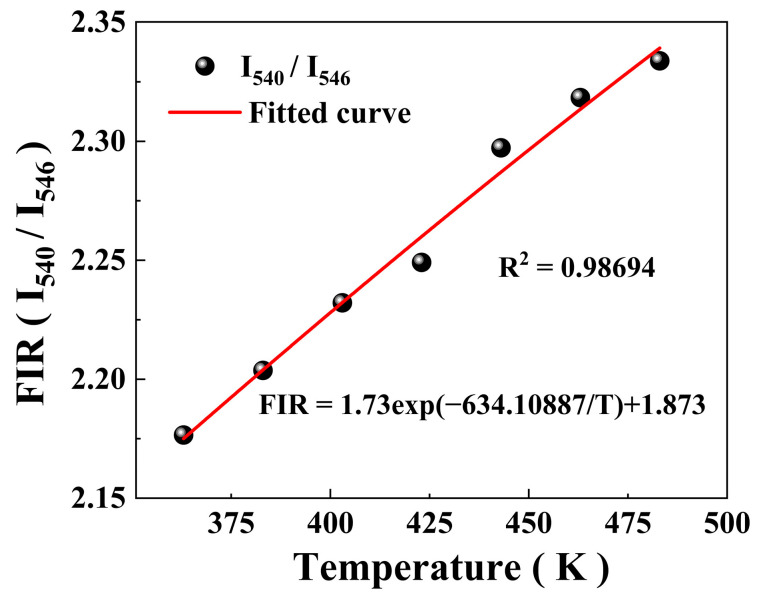
Fluorescence intensity ratio (FIR) changes and response to high temperature.

The thermal population of the ^5^F_4_ and ^5^S_2_ energy levels of Ho^3+^, and the luminescence intensity follows the Boltzmann distribution law, as expressed in Equation (5) [21]:

(5) $$\mathrm{FIR}=\frac{I_{540}}{I_{546}}=A\exp(-\frac{\Delta E}{k_{B}T})+B,$$

where *A* is a constant, *I*_540_ and *I*_546_ are integral intensities, ΔE is the energy level gap, $k_{B}$ is the Boltzmann constant, *T* denotes the absolute temperature, and *B* is a correction factor. This law was used to analyse the overlap of the emission peaks. The fitting results of the FIR spectra are shown in Figure 9. *R*^2^ signifies the degree of discrepancy between the theoretical fit and experimental data. The intensity ratio of the UC luminescence increased as the temperature increased; *R*^2^ in Figure 9 was 0.98694, indicating a high fit correlation.

The energy gap value at high temperatures is 440 cm^−1^; this value deviates considerably from that reported in the literature (180 cm^−1^), possibly because the energy gap at the ^5^F_4_/^5^S_2_ level is small, a large overlap exists between the two emission bands, and the lattice vibration is intense at high temperatures, which leads to significant inhomogeneous broadening of the spectral lines. The impact of the incoherent superposition of the two emission bands is pronounced, which significantly affects the thermal coupling effect, resulting in deviations from the actual data. Moreover, thermal quenching occurred. With increasing temperature, the radiation-free relaxation increases, and energy is dissipated as heat.

**Figure 10:**
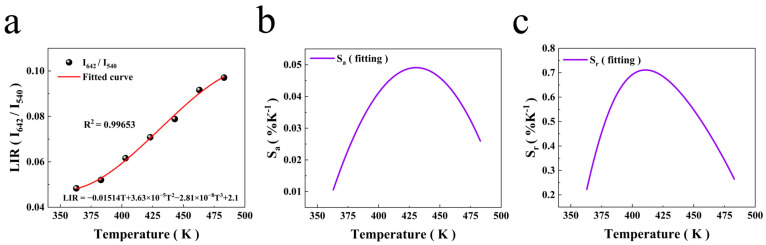
(**a**) Luminescence intensity ratio (LIR) changes and response to temperature; (**b**) absolute sensitivity to temperature; (**c**) relative sensitivity to temperature.

The ^5^F_4_ and ^5^F_5_ levels of Ho^3+^ belong to a pair of non-thermally coupled levels, and the energy level interval between them was calculated to be approximately 3000 cm^−1^. Therefore, temperature sensing at non-thermally coupled levels can be realised using the LIR of the UC green and red luminescence bands of NaLuF_4_:20%Yb^3+^/2%Ho^3+^ micron-sized crystals, which change with temperature. The formula used is as follows [22]:

(6) $$\mathrm{LIR}=\frac{I_{540}}{I_{642}}=CT+DT^{2}+ET^{3}+F,$$

where *C*, *D*, *E*, and *F* are constants; *I*_540_ and *I*_642_ are integral intensities; and *T* denotes the absolute temperature. The fitting results of LIR are shown in Figure 10a. The experimental data fit this line well, and the deviation *R*^2^ = 0.99653. The spectrum shows that the luminescence intensity at 540 nm decreased significantly with increasing temperature, whereas the luminescence intensity at 642 nm gradually decreased. The difference in the response of the non-thermally coupled levels to temperature is conducive to obtaining a higher temperature sensitivity. S*_a_* and S*_r_* are also crucial metrics for evaluating temperature sensors [11].

(7) $$S_{a}=\left|\frac{d\mathrm{FIR}}{dT}\right|=\left|C+2DT+3ET^{2}\right|,$$

(8) $$S_{r}=\frac{1}{\mathrm{LIR}}\left|\frac{d\mathrm{LIR}}{dT}\right|.$$

The S*_a_* and S*_r_* of the 540 and 642 nm non-thermally coupled energy levels at high temperatures were calculated using Equations (7) and (8). Figure 10b,c shows the absolute sensitivity of the NaLuF_4_:20%Yb^3+^/2%Ho^3+^ micron-sized crystal applied to LIR temperature measurement technology upon stimulation with a 980 nm laser. The variable initially increased before subsequently declining as the temperature increased. At 428 K, S*_a_* reached its maximum value of 0.049% K^−1^. The relative sensitivity initially increased and then decreased as the temperature increased. At 408 K, S*_r_* had a maximum value of 0.71% K^−1^. As shown in Table 1, comparing several typical thermometric materials, the relative sensitivity of Yb^3+^/Ho^3+^-doped NaLuF_4_ surpasses that of most materials documented in the literature. The results indicate that NaLuF_4_:20%Yb^3+^/2%Ho^3+^ micron-sized crystals have excellent temperature sensing performance under high-temperature conditions and have potential applications in remote non-contact temperature sensing.

The absolute sensitivity S*_a_* and relative sensitivity S*_r_* are important indicators for evaluating temperature sensors. The greater the magnitude of the S value, the more pronounced the variation of FIR values with temperature. S*_a_* can be expressed as follows [30]:

(9) $$S_{a}=\left|\frac{d\mathrm{FIR}}{dT}\right|=\left|A\exp(-\frac{\Delta E}{k_{B}T})(\frac{\Delta E}{k_{B}T^{2}})\right|,$$

**Figure 11:**
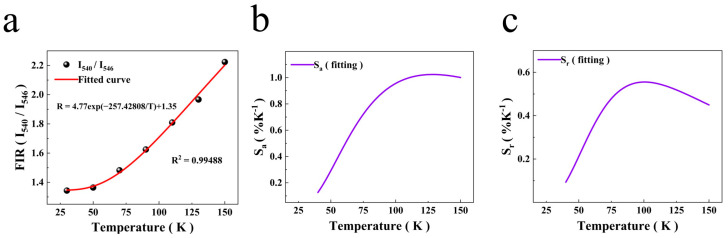
(**a**) FIR changes and response to low temperature; (**b**) the relationship between S*_a_* and temperature; (**c**) the relationship between S*_r_* and temperature.

S*_r_* can be articulated as follows [31]:

(10) $$S_{r}=\frac{1}{\mathrm{FIR}}\left|\frac{d\mathrm{FIR}}{dT}\right|=\left|\frac{\Delta E}{k_{B}T^{2}}\right|,$$

the fitting results of FIR at low temperatures are shown in Figure 11a. The FIR increased with temperature, and *R*^2^ in Figure 11a is 0.99488, indicating a very high fit correlation. The S*_a_* and S*_r_* of the 540 and 546 nm thermally coupled energy levels at low temperatures were calculated using Equations (9) and (10). S*_a_* and S*_r_* increased significantly at low temperatures. The overall trend exhibited an initial ascent followed by a subsequent descent. The maximum value of S*_a_* was 1.02% K^−1^ at 125 K, and that of S*_r_* were 0.55% K^−1^ at 100 K, respectively. This shows that the sample has better temperature sensing performance at low temperatures. The energy gap value at low temperatures is 178.8 cm^−1^, which is similar to that reported in the literature, 180 cm^−1^. A comparison of previously reported results with the research results of this work (as shown in Table 2) reveals that Yb^3+^/Ho^3+^-co-doped NaLuF_4_ based on thermally coupled energy levels is an excellent temperature sensing material.

**Figure 12:**
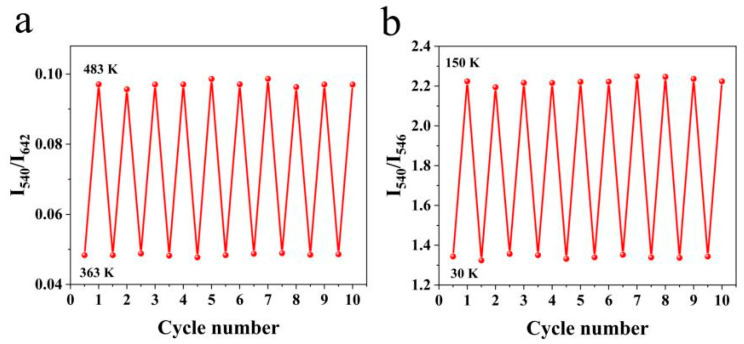
(**a**) Temperature cycling measurement of NaLuF_4_:Yb^3+^/Ho^3+^ between 363 K and 483 K; (**b**) Temperature cycling measurement of NaLuF_4_:Yb^3+^/Ho^3+^ between 30 and 150 K.

The repeatability is of great significance to the practical application of optical temperature measurement. As shown in Figure 12a,b, after continuously warming and cooling the system between 363 and 483 K, the LIR value was found to be stable. Within the temperature range of 30–150 K, employing the same methodology, the FIR value remained stable. In repetitive experiments, it was found that the percentage deviation of LIR and FIR does not exceed 1.5%. The temperature uncertainty can be determined by the following [14]:

(11) $$\delta T=\frac{1}{S_{r}}\frac{\delta R}{R}$$

Assuming a practical limit of δR/R = 0.5% for commercial detectors [36], a low temperature uncertainty of approximately 0.9 K can be obtained at 100 K. Under high-temperature conditions, LIR exhibits an uncertainty of 0.7 K at 408 K. The values are similar to those reported in the literature [36,37].

## 4. Conclusions

In this study, NaLuF_4_:Yb^3+^/Ho^3+^ micron-sized crystals with high UC luminescence intensities were synthesised using a hydrothermal synthesis method. The micro-level morphology, spectral characteristics, and thermal sensing characteristics of the particles were examined. The UC luminescence properties of the samples were studied at low temperature. In the UC spectrum, the ^5^F_4_/^5^S_2_→^5^I_8_ and ^5^F_5_→^5^I_8_ transitions corresponded to the main emission peaks. As the temperature increased, the intensity of the green emission gradually decreased. G1 strengthened and G2 weakened. We then studied the dependence of the UC luminescence intensity on the excitation power and the variation in the lifetime with temperature. The experiment revealed that the green emission intensity depends strongly on the temperature; therefore, we studied the sensing performance of the sample at low temperature. FIR and LIR methods were used to determine the temperature dependence of thermally coupled levels, ^5^F_4_ and ^5^S_2_, and non-thermally coupled levels, ^5^F_4_ and ^5^F_5_. The maximum sensitivity was 1.02% K^−1^ at 125 K at low temperatures. The non-thermally coupled levels, ^5^F_4_ and ^5^F_5_, had a maximum sensitivity of 0.71% K^−1^ at 408 K. Yb^3+^/Ho^3+^-co-doped NaLuF_4_ micron-sized materials have significant potential for optical temperature sensing applications.

## Figures and Tables

**Table 1:** Comparison of temperature sensing performance based on Ho^3+^ non-thermal coupling level.

Sample	Energy Level	Temperature/K	S*_a_*_,*max*_/%K^−1^	S*_r_*_,*max*_/%K^−1^	Ref.
Sr_3_Y_0.88_(PO_4_)_3_:Yb^3+^/Ho^3+^	^5^F_4_/^5^F_5_→^5^I_8_	298–573	0.42	0.16	[23]
Y_2_O_3_:Yb^3+^/Ho^3+^/Ge^2+^	^5^F_4_/^5^F_5_→^5^I_8_	300–400	0.52	0.62	[24]
Y_2_O_3_:Yb^3+^/Ho^3+^	^5^F_4_/^5^F_5_→^5^I_8_	293–873	3.08	0.38	[25]
NaLuF_4_:Yb^3+^/Ho^3+^	^5^F_4_/^5^F_5_→^5^I_8_	390–780	0.08	0.83	[26]
Y_2_O_3_:Yb^3+^/Ho^3+^/Zn^2+^	^5^F_4_/^5^F_5_→^5^I_8_	300–673	0.30	0.23	[27]
NaLuF_4_:Yb^3+^/Ho^3+^/Ce^3+^	^5^F_4_/^5^F_5_→^5^I_8_	303–573	4.37	-	[13]
Ca_3_La_6_Si_6_O_24_:Yb^3+^/Ho^3+^	^5^F_4_/^5^F_5_→^5^I_8_	293–573	-	0.0015	[28]
Pb(Mg_1/3_Nb_2/3_)O_3_–PbTiO_3_:Yb^3+^/Ho^3+^	^5^F_4_/^5^F_5_→^5^I_8_	298–553	-	0.0077	[29]
NaLuF_4_:Yb^3+^/Ho^3+^	^5^F_4_/^5^F_5_→^5^I_8_	363–483	0.049	0.71	This work

**Table 2:** Comparison of temperature sensing performance based on Ho^3+^ thermal coupling level.

Sample	Energy Level	Temperature/K	S*_a_*_,*max*_/%K^−1^	S*_r_*_,*max*_/%K^−1^	Ref.
Ba_3_Y_4_O_9_:Yb^3+^/Ho^3+^/Tm^3+^	^5^F_4_/^5^S_2_→^5^I_8_	298–573	0.18	-	[32]
BaWO_4_:Yb^3+^/Ho^3+^/Gd^3+^	^5^F_4_/^5^S_2_→^5^I_8_	303–473	0.203	-	[33]
In–Zn–Sr–Ba:Ho^3+^/Er^3+^	^5^F_4_/^5^S_2_→^5^I_8_,^5^I_7_	20–300	-	0.36	[34]
NaLuF_4_:Yb^3+^/Ho^3+^	^5^F_4_/^5^S_2_→^5^I_8_	390–780	0.14	0.83	[26]
Y_2_O_3_:Yb^3+^/Ho^3+^	^5^F_4_/^5^S_2_→^5^I_8_	300–500	0.52	-	[35]
Gd_2_O_3_:Ho^3+^/Yb^3+^/Li^+^	^5^F_4_/^5^S_2_→^5^I_8_	300–623	0.92	-	[5]
NaLuF_4_:Yb^3+^/Ho^3+^	^5^F_4_/^5^S_2_→^5^I_8_	30–150	1.02	0.55	This work

## Data Availability

Data is contained within the article.
